# Photoinactivation of Planktonic Cells, Pseudohyphae, and Biofilms of *Candida albicans* Sensitized by a Free-Base Chlorin and Its Metal Complexes with Zn(II) and Pd(II)

**DOI:** 10.3390/antibiotics12010105

**Published:** 2023-01-06

**Authors:** Paula V. Cordero, María G. Alvarez, Edwin J. Gonzalez Lopez, Daniel A. Heredia, Edgardo N. Durantini

**Affiliations:** IDAS-CONICET, Departamento de Química, Facultad de Ciencias Exactas, Físico-Químicas y Naturales, Universidad Nacional de Río Cuarto, Ruta Nacional 36 Km 601, Río Cuarto X5804BYA, Argentina

**Keywords:** antifungal, photoinactivation, chlorin, *Candida albicans*, pseudohyphae, biofilm

## Abstract

Invasive candidiasis is an important cause of morbidity and mortality, and its occurrence is increasing due to the growing complexity of patients. In particular, *Candida albicans* exhibits several virulence factors that facilitate yeast colonization in humans. In this sense, the photodynamic inactivation of yeasts is a promising new alternative to eliminate fungal infections. Herein, the photodynamic activity sensitized by a free-base chlorin (TPCF_16_) and its complexes with Zn(II) (ZnTPCF_16_) and Pd(II) (PdTPCF_16_) was investigated in order to eliminate *C. albicans* under different forms of cell cultures. A decrease in cell survival of more than 5 log was found in planktonic cells incubated with 5 μM TPCF_16_ or ZnTPCF_16_ upon 15 min of white-light irradiation. The mechanism of action mainly involved a type II pathway in the inactivation of *C. albicans* cells. In addition, the photodynamic action induced by these chlorins was able to suppress the growth of *C. albicans* in a culture medium. These photosensitizers were also effective to photoinactivate *C. albicans* pseudohyphae suspended in PBS. Furthermore, the biofilms of *C. albicans* that incorporated the chlorins during the proliferation stage were completely eradicated using 5 μM TPCF_16_ or ZnTPCF_16_ after 60 min of light irradiation. The studies indicated that these chlorins are effective photosensitizing agents to eliminate *C. albicans* as planktonic cells, pseudohyphae, and biofilms.

## 1. Introduction

Fungal infections are becoming a major health problem, causing high mortality rates, medical costs for treatments, and an increase in hospitalized patients [1]. The incidence of fungal skin infections is continuously increasing all over the world, representing a great challenge for health care. In addition, hospital-acquired fungal infections are constantly increasing and are considerably problematic for immunocompromised patients [2]. In general, cutaneous and subcutaneous fungal diseases are caused by pathogenic or opportunistic organisms. In this sense, *Candida albicans* is one of the most important human pathogenic fungi [3]. This microorganism causes millions of cutaneous, mucosal, and systemic diseases that can be life-threatening. The establishment of fungal infections is mediated by virulence factors such as the yeast–hyphal transition and formation of biofilm [4]. Hyphae are necessary during infections in order to penetrate epithelial cell walls. Furthermore, biofilm production facilitates widespread hyphal growth and acts to cause invasive fungal diseases [5]. Therefore, biofilm formation represents a major virulence factor during candidiasis. Different biological media are capable of supporting *C. albicans* biofilm formation. Thus, the incidence of candidiasis increased with the increased use of medical devices in clinical practice [6]. In addition, these infections are aggravated by the appearance of yeasts resistant to conventional antifungal treatments [7,8]. This leads to the need to develop new antifungal therapies that can improve clinical outcomes for people with life-threatening fungal diseases worldwide [9]. In recent years, several antimicrobial strategies were planned to eliminate fungal infections and to decrease the development of virulence factors [10].

For this purpose, the photodynamic inactivation (PDI) of yeasts represents an interesting alternative to treat fungal diseases or reduce the formation of virulence factors [11]. This therapy is based on the administration of a photosensitizing agent (PS) that is rapidly bound to microorganisms. Selective irradiation with an adequate wavelength in the visible region in aerobiosis leads to the generation of reactive oxygen species (ROS), which can then react with biomolecules to cause loss of functionality and, consequently, cell inactivation [12]. In this procedure, the triplet-excited state of the PS (^3^PS*) can interact with cell components by electron transfer or hydrogen abstraction to form free-radicals. In addition, these intermediates can react with ground state molecular oxygen (O_2_(^3^∑^-^_g_)) to produce superoxide anion radicals, hydroxyl radicals, and hydrogen peroxide by a type I pathway [13]. On the other hand, ^3^PS* can generate singlet molecular oxygen (O_2_(^1^Δ_g_)) by energy transfer to O_2_(^3^∑^-^_g_) through a type II process [11,12]. Both mechanisms can act simultaneously, and the prevalence of one of them can depend mainly on the characteristics of the PS, the substrates, and the microenvironmental polarity [14]. Until now, PDI studies have not shown evidence of the development of microbial resistance related to the application of this therapy [15]. Therefore, this methodology represents a promising alternative for the elimination of fungal cells.

**Figure 1:**
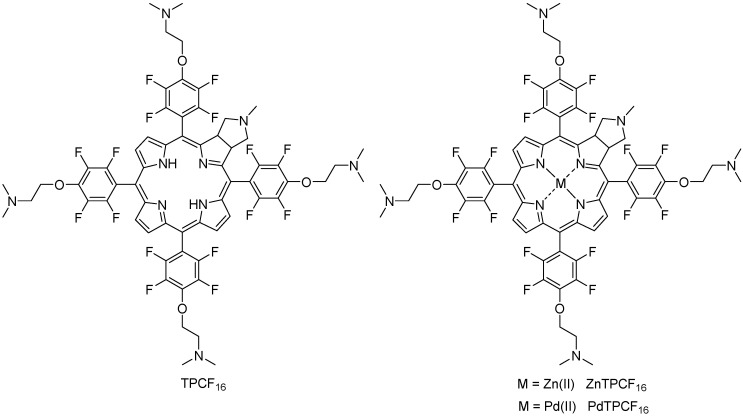
Molecular structure of TPCF_16_ and its complexes with Zn(II) and Pd(II).

In particular, porphyrin derivatives and their metal complexes have presented interesting applications as PSs against human microbial pathogens through PDI [16,17]. In this study, we investigate the photodynamic effect induced by a chlorin derivative (TPCF_16_) and its complexes with Zn(II) (ZnTPCF_16_) and Pd(II) (PdTPCF_16_) (Figure 1) as PSs to eliminate *C. albicans*. Due to the background with chlorin derivatives, the compounds selected as PSs in this work may present an appropriate role as antifungal agents using photodynamic inactivation treatments. It was previously demonstrated that the free-base chlorin TPCF_16_ was an efficient PS to produce a high decrease in the cell viability of bacterial cells [18]. Furthermore, cationic chlorins derived of 5,10,15,20-tetrakis-(pentafluorophenyl)-2,3-[methano(*N*-methyl)iminomethano]chlorin (TPCF_20_) exhibited high PDI efficacy towards planktonic and biofilm forms of *E. coli* [19,20]. Herein, the spectroscopic properties and photodynamic activity of TPCF_16_, ZnTPCF_16_, and PdTPCF_16_ were established in homogenic solutions. Furthermore, the photoinactivation ability sensitized by these chlorins was evaluated in *C. albicans* under different cell culture conditions. First, photokilling was tested in planktonic yeast cell suspensions. Insights into the predominant mechanism of photodynamic action were established in cell suspensions using different ROS scavengers. In addition, the photoinactivation activity induced by these chlorins was determined with the growth of *C. albicans* in culture broth. Considering that reversible cell morphogenesis is an important virulence factor, these compounds were also assayed to eliminate *C. albicans* pseudohyphae. Lastly, the photoinactivating capacities of the chlorins were investigated in *C. albicans* biofilms for the prevention and control of these clinical microbial communities.

## 2. Materials and Methods

Details of materials and instrumentation are available in Supplementary Materials.

### 2.1. Spectroscopic Measurements

UV-visible absorption and fluorescence spectra were attained in a quartz cell of 1 cm path length at room temperature and by using *N*,*N*-dimethylformamide (DMF) as an organic solvent. Emission spectra were measured by exciting the samples at 408 nm. At this wavelength, the absorbances of the samples were approximately 0.05. The emission spectra were recorded and integrated in the range between 600 and 800 nm. The fluorescence quantum yields (Φ_F_) of the chlorins were determined by comparing the area of the emission spectrum for each PS with that of 5,10,15,20-tetra(4-methoxyphenyl)porphyrin (TMP), which was used as a reference (Φ_F_ = 0.14) [21]. Excitation spectra were measured by following the fluorescence emissions of the chlorins at 700 nm.

### 2.2. Photooxidation of DMA

Samples containing 9,10-dimethylanthracene (DMA, 35 μM) and chlorin (A = 0.1 at 610 nm) in 2 mL of DMF were irradiated with light at λ_irr_ = 610 nm in a quartz cell of 1 cm path length. The photodecomposition of DMA was evaluated by the decrease in absorbance at λ_max_ = 379 nm (Supplementary Figure S1). The values of the observed rate constants of DMA photo-oxidation (*k*_obs_^DMA^) were determined by a linear least-squares fit of the pseudo-first-order kinetic plots of ln(A_0_/A) vs. time. The quantum yields of O_2_(^1^Δ_g_) production (Φ_Δ_) were determined by comparing the *k*_obs_^DMA^ values for the corresponding chlorins with that of methylene blue (MB, Φ_Δ_ = 0.52), which was used as a reference [22].

### 2.3. Strains and Cultures of C. albicans

The *C. albicans* (PC31) strain was previously recognized and characterized [23]. Yeast cells were grown aerobically overnight in 4 mL Sabouraud broth at 37 °C until the stationary phase. The cells were collected via centrifugation of the culture broths (1200× *g* for 15 min). Then, the yeast cells were resuspended in phosphate-buffered saline (PBS, 4 mL, 10 mM, pH = 7.2) to harvest a cell suspension of ~10^7^ colony forming units (CFU)/mL. After that, cultures were diluted 1/10 in PBS to obtain ~10^6^ CFU/mL [24].

### 2.4. Experiments in C. albicans Planktonic Cells

Assays were attained using 2 mL cell suspensions of ~10^6^ CFU/mL in PBS, which were placed in Pyrex-brand culture tubes (13 × 100 mm). Yeast cells were incubated with 1 and 5 μM PS for 30 min in the dark at 37 °C. Chlorins were added from the stock solution to a volume of 0.5 mM in DMF. The amount of DMF used did not exceed 1% *v*/*v*, and this quantity of organic solvent was not toxic to *C. albicans* cells.

In the studies of the photodynamic mechanism of action, the following tests were performed separately: *C. albicans* cell suspensions (2 mL, ~10^6^ CFU/mL) in PBS were treated with 50 mM of (1) sodium azide (50 mM), (2) diazabicyclo [2.2.2]octane (DABCO, 50 mM), (3) D-mannitol (50 mM), or (4) L-cysteine (50 mM), of which 1 M each was added from stock solutions into water [24,25]. Each cell culture was incubated for 30 min at 37 °C in the dark. Subsequently, 1 μM chlorine was added to each culture tube, and they were kept for another 30 min in the dark at 37 °C. Studies in deuterated water (D_2_O) were performed using cell suspensions (2 mL, ~10^6^ CFU/mL) in PBS, which were centrifuged (1200× *g* for 15 min) and resuspended in D_2_O (2 mL). Then, the cell suspensions in D_2_O were incubated with 1 μM PS for 30 min in the dark at 37 °C.

In all experiments, 200 µL of cell suspensions were placed in wells of a 96-well microtiter plate, and they were irradiated with white light (90 mW/cm^2^). *C. albicans* cells were quantified by performing serial dilution in PBS and then counting with the spread plate method. Viable *C. albicans* cells were determined on Sabouraud agar plates after incubation for 48 h at 37 °C [22,24].

### 2.5. Growth Curves of C. albicans

Cultures of *C. albicans* cells were grown overnight as previously described [26]. An aliquot (1 mL) of yeast culture was transferred to 20 mL of Sabouraud broth in PBS. The cell suspension was homogenized, and portions of 2 mL each were treated with 5 µM PS in Pyrex brand culture tubes (13 × 100 mm). The tubes were continuously irradiated with white light (90 mW/cm^2^) at 37 °C. The growth of *C. albicans* cells was evaluated by analyzing spectroscopic determinations at 660 nm. The values at this wavelength were adjusted by subtracting the absorbance due to the chlorin.

### 2.6. Studies in C. albicans Pseudohyphae

*C. albicans* cells (~10^6^ CFU/mL) were incubated in human serum (HS) for 4 h at 37 °C to produce the formation of pseudohyphae [27]. After this incubation period, the generation of germ tubes was established with optical microscopy [28]. Pseudohyphae were harvested via centrifugation (1200× *g* for 15 min) and suspended in PBS. Then, 2 mL of pseudohyphae suspension was placed in Pyrex brand culture tubes (13 × 100 mm). *C. albicans* pseudohyphae were incubated with 1 and 5 µM chlorin for 30 min in the dark at 37 °C. Culture aliquots (200 µL) were placed in wells of a 96-well microtiter plate and irradiated with white light (90 mW/cm^2^) for different periods of time (2, 5, 15 and 30 min). Viable *C. albicans* pseudohyphae were determined as reported [28].

### 2.7. Tests in C. albicans Biofilms

*C. albicans* cell suspension (~10^7^ CFU/mL) was prepared in PBS supplemented with 7% fetal bovine serum (FBS). Then, 900 µL of this culture was placed in the wells of a 48-well microtiter plate containing a polyvinylchloride (PVC) disc (5mm Ø × 1.5 mm) in each well. For the adhesion step, the plate was incubated for 90 min at 37 °C with shaking (75 r.p.m.). After that, each disc was removed and washed twice with PBS to remove non-adhered cells. In the proliferation step, the PVC discs in the 48-well microtiter plate were treated with 5 μM chlorin in Sabouraud broth supplemented with 7% FBS and incubated for 18 h at 37 °C. After that, the discs were washed with PBS and covered with 900 mL of PBS in the wells. The biofilms were irradiated with white light for 60 min. Then, each disc and the well contents were placed in a test tube, sonicated for 1 min, and vigorously vortexed for 2 min to detach cells from the biofilms on the disc. Viable *C. albicans* cells were determined as explained above.

### 2.8. Controls and Statistical Analysis

Controls of yeast cells were performed in the absence and presence of chlorin in the dark. In addition, controls were carried out in irradiated cells with white light (90 mW/cm^2^) in the absence of chlorin. In all *C. albicans* photoinactivation experiments, the temperature was maintained at 37 °C. This temperature did not affect the viability of the cells in the absence of PSs. Each experiment was repeated separately three times, and the values were achieved in triplicate. The significance of the differences between the results was established using one-way ANOVA with a confidence level of 95% [25]. Data in the plots were denoted as the mean ± standard deviation.

## 3. Results and Discussion

### 3.1. Molecular Structure of Chlorin Derivatives

The molecular structures of the chlorins are shown in Figure 1. These PSs contain five tertiary amine groups, one in the *N*-methylpyrrolidine substituent (*p*K_a_~10.46) and four in the 3-(*N*,*N*-dimethylamine)propanol (*p*K_a_~9.51) groups [28,29]. Considering the *p*K_a_ values, these basic amine substituents are precursors of cationic groups by protonation in an aqueous medium at physiological pH. Although pKa values are not definitive proof of protonation, similar photoinactivation was previously found between a cationic porphyrin and its analogue without intrinsic charges substituted by basic amine groups [26]. Furthermore, four of them are covalently attached to the chlorin structure by a two-carbon aliphatic chain. This linker provides mobility to the positively charged precursor groups. Furthermore, this spacer prevents changes in the spectroscopic and photodynamic properties of the chlorin because the amine groups are not conjugated to the macrocycle [24,26]. Both the formation of cationic groups on the periphery of the chlorin macrocycle and their mobility can allow a better interaction of PS with yeast, thus increasing the photoinactivating capacity of *C. albicans* cells [26]. Furthermore, complexation of TPCF_16_ with Zn(II) and Pd(II) was performed because these metals form stable complexes with tetrapyrrole macrocycles with a 1:1 metal-to-ligand molar ratio [30,31]. Pd(II) complexes are characterized by a typical four-coordinate metal center, whereas Zn(II) complex centers are mainly five-coordinate [32]. In TMP, the Zn(II) lies on an inversion center and is coordinated in an almost ideal square planar geometry [33]. The asymmetric unit also contains one solvent molecule. Complexation with Zn(II) and Pd(II) was used to modify the photodynamic properties of the free-base chlorin because these metals each produce a significant heavy-atom effect [30]. This phenomenon enhances the intersystem crossing of a PS to ^3^PS* by spin–orbit coupling [31]. Therefore, the introduction of heavy atoms can result in an increase of ROS generation [31,34,35].

### 3.2. Spectroscopic Characterization

**Figure 2:**
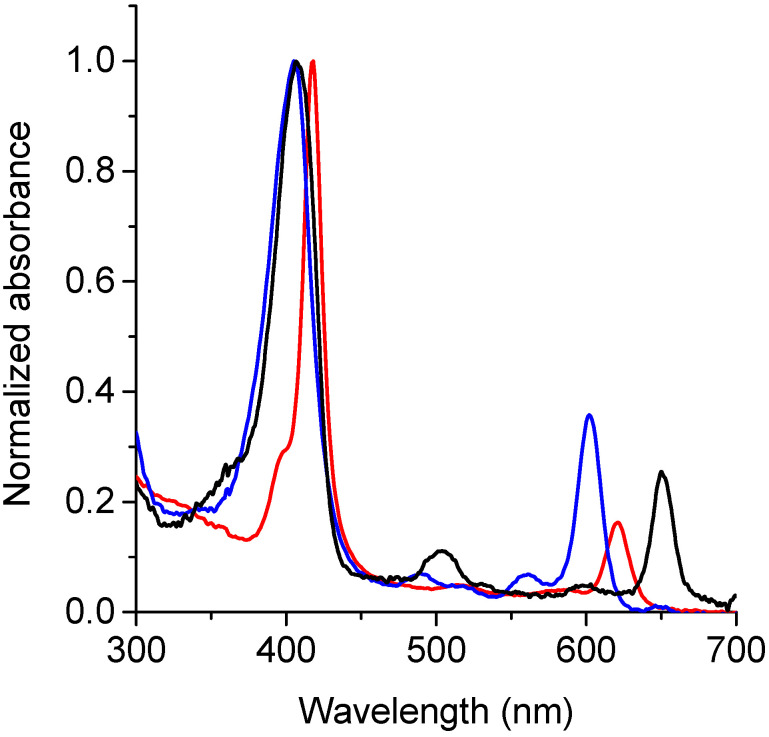
Absorption spectra of TPCF_16_ (black line), ZnTPCF_16_ (red line), and PdTPCF_16_ (blue line) in DMF.

UV-visible absorption spectra of TPCF_16_, ZnTPCF_16_, and PdTPCF_16_ were determined in DMF. As shown in Figure 2, the spectra are characterized by a high intensity Soret band around 405–418 nm. Similar absorbance maxima were found for TPCF_16_ and PdTPCF_16_, whereas the Zn(II) complex showed a bathochromic shift of 10 nm compared to the free-base chlorin. These absorption peaks presented molar absorption coefficients (ε) in the order of 10^5^ Lmol^−1^cm^−1^ (Table 1). Furthermore, these chlorins exhibited Q bands of lower intensity in the visible region between 500 and 700 nm. All spectra showed an intense Q(0-0) band that had ε values of about 10^4^ Lmol^−1^cm^−1^ (Table 1), typical of chlorin derivatives [18,36]. This band is hypsochromically shifted with respect to the free-base chlorin by 30 and 49 nm for ZnTPCF_16_ and PdTPCF_16_, respectively. Similar results were previously found for metal complexes of the chlorins’ derivatives [31,32,33,34,35].

**Figure 3:**
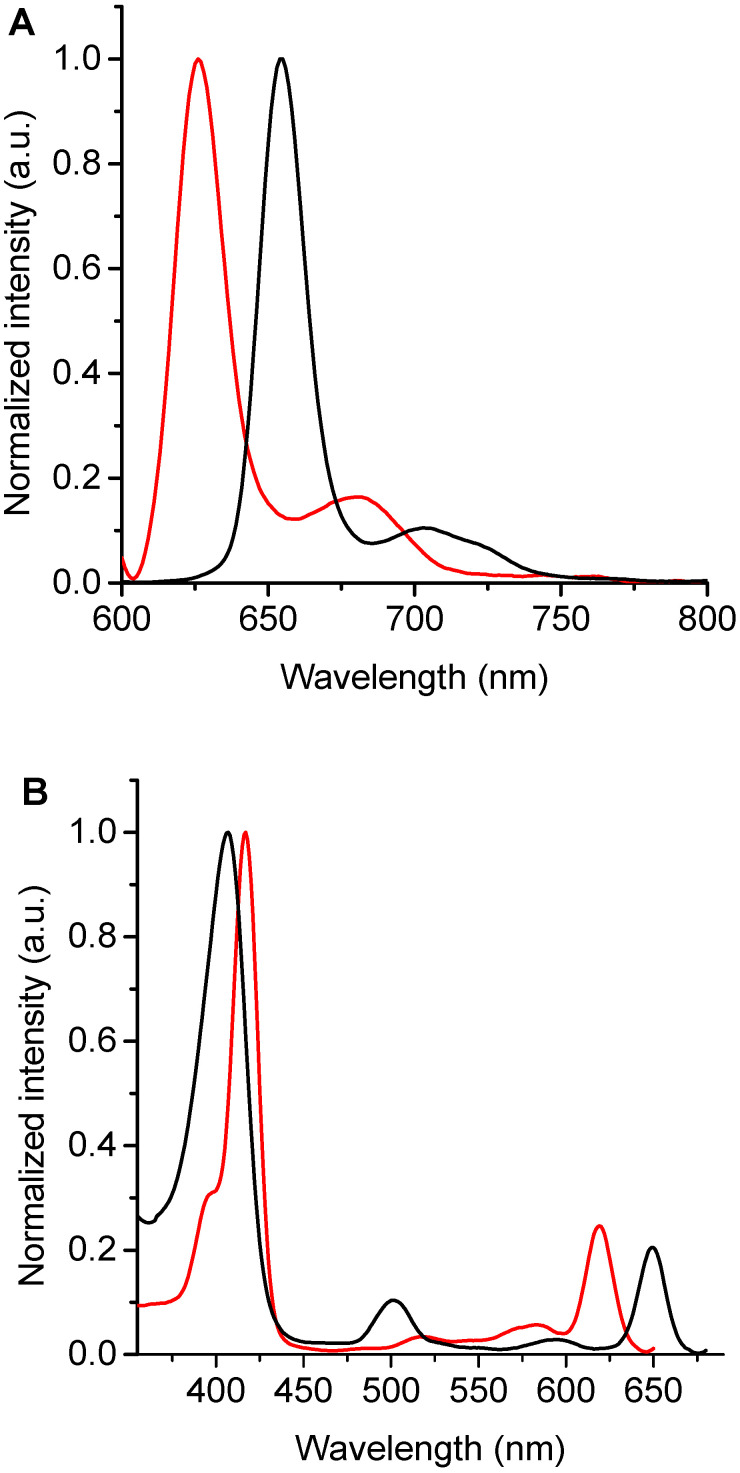
(**A**) Fluorescence emission spectra (λ_exc_ = 408 nm) and (**B**) fluorescence excitation spectra (λ_em_ = 700 nm) of TPCF_16_ (black line) and ZnTPCF_16_ (red line) in DMF.

Fluorescence emission spectra of these chlorins were recorded in DMF (Figure 3A). The spectra of TPCF_16_ and ZnTPCF_16_ showed two bands in the red spectral region, with a main emission band of Zn(II) complex hypsochromically shifted by 27 nm compared to free-base chlorin (Table 1). These fluorescence emission bands are typical for similar chlorin derivatives [18]. These bands can be assigned to the electronic transitions that take place from the first singlet excited state of the chlorin macrocycle to the first two vibrational levels of the ground state, which are denoted as Q(0–0) and Q(0–1), respectively [36]. In contrast, the emission of PdTPCF_16_ was negligible under these conditions. This singlet excited state quenching effect was previously observed in Pd(II) complexes with tetrapyrrole macrocycles [30]. Furthermore, Stokes shifts for TPCF_16_ and ZnTPCF_16_ were determined from the wavelengths of the last absorption band and the first emission band, Q(0–0), giving values of 2 and 4 nm, respectively. This behavior indicates that the absorption energy in the first excited singlet state is very similar to its relaxation energy [18,36]. Therefore, the structural changes between the ground and excited states of these molecules are minimal, mainly as a consequence of the rigidity of the chlorin macrocycle. The Φ_F_ values of TPCF_16_ and ZnTPCF_16_ were obtained in DMF (Table 1), using TMP as a reference [21]. Free-base chlorin presented a Φ_F_ value that agreed with those reported for similar chlorin derivatives [18]. As expected, this Φ_F_ value decreased in the complex with Zn(II) due to the heavy-atom effect [30]. These Φ_F_ values are appropriate to determine interactions of these chlorins with microorganisms and to carry out studies using fluorescence microscopy techniques in *C. albicans* cells [37].

Moreover, fluorescence excitation spectra of TPCF_16_ and ZnTPCF_16_ were recorded in DMF by measuring their emissions at 700 nm (Figure 3B). This methodology allows differentiation of the Q bands of the chlorins in detail, mainly when these compounds are present in very low concentrations. Furthermore, when chlorins are bound to yeast cells, where the absorption spectrum may overlap with that of other chromophores, the excitation spectrum is important for observation of the exact shapes and positions of the PS absorption bands [23]. Both chlorins showed a close similarity between the fluorescence excitation spectra and the corresponding absorption spectra (Figure 2 and Figure 3B). Therefore, these results confirm that TPCF_16_ and ZnTPCF_16_ were mainly dissolved as monomers in this organic solvent.

### 3.3. Photooxidation of DMA and O_2_(^1^Δ_g_) Formation

**Figure 4:**
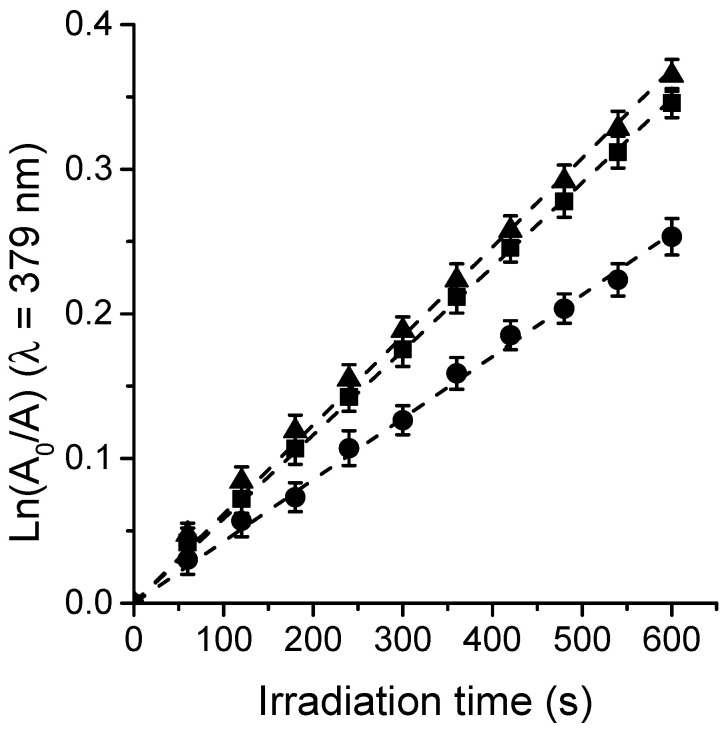
Kinetic plot for the decomposition of DMA (35 μM) photosensitized by ZnTPCF_16_ (■), PdTPCF_16_ (▼), and the reference MB (●) in DMF; λ_irr_ = 610 nm.

O_2_(^1^Δ_g_) represents one of the main cytotoxic species involved in the photoinactivation of microorganisms [12,15]. Thus, the generation of O_2_(^1^Δ_g_) sensitized by TPCF_16_, ZnTPCF_16_, and PdTPCF_16_ was measured in air-equilibrated solutions of DMF. In these studies, DMA was used as a molecular probe, which quenched O_2_(^1^Δ_g_) primarily through a chemical reaction to produce the 9,10-endoperoxide derivative [38]. Photooxidation of DMA upon reaction with O_2_(^1^Δ_g_) was observed following the decrease in absorbance of the quencher at 379 nm (Figure S1) [36]. The DMA absorption band decreased progressively in the presence of chlorin when the solution was exposed to light at 610 nm, indicating that these PSs generated O_2_(^1^Δ_g_). It is important to note that during the experiments, the UV-visible spectra of the chlorins remained without significant changes, indicating that these compounds were stable to photobleaching during these measurements. Figure 4 shows that the reactions followed a pseudo-first-order kinetic for DMA decomposition. The values of the *k*_obs_^DMA^ were obtained from the slopes of the linear fits of the data (Table 2). To determine the Φ_Δ_ values, the kinetic results were compared with those attained for MB, which was used as a reference [22]. As can be seen in Figure 4, the formation of O_2_(^1^Δ_g_) sensitized by both chlorin metal complexes was achieved at similar rates. Therefore, comparable Φ_Δ_ values were obtained for ZnTPCF_16_ and PdTPCF_16_ (Table 2). Similar improvements of Φ_Δ_ values with respect to free-base chlorin were previously reported for chlorin derivatives forming complexes with Zn(II) and Pd(II) in DMF [34,39]. While TPCF_16_ showed a lower Φ_Δ_ value, its Zn(II) and Pd(II) complexes were found to be more effective in forming O_2_(^1^Δ_g_) in organic solvent due to the heavy-atom effect. These results reveal that these chlorins are capable of undergoing a type II photomechanism producing O_2_(^1^Δ_g_) under irradiation.

### 3.4. Photokilling of C. albicans Planktonic Cells

**Figure 5:**
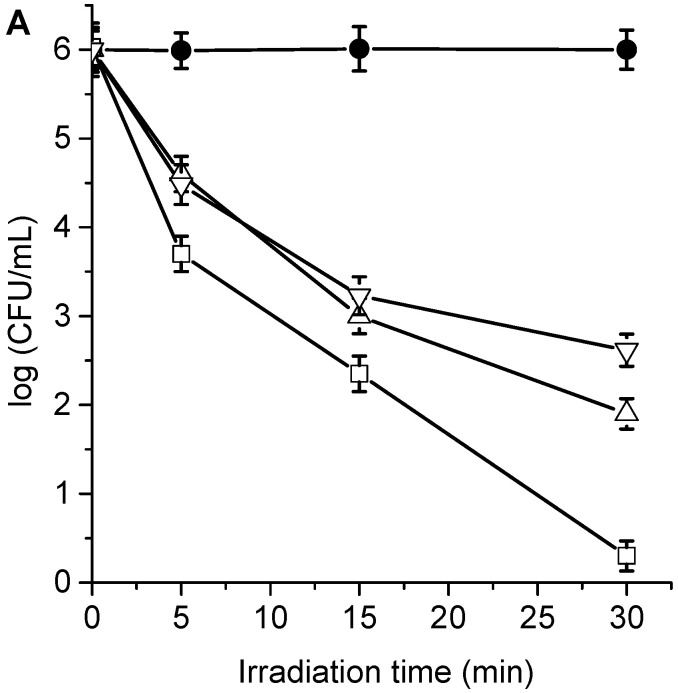

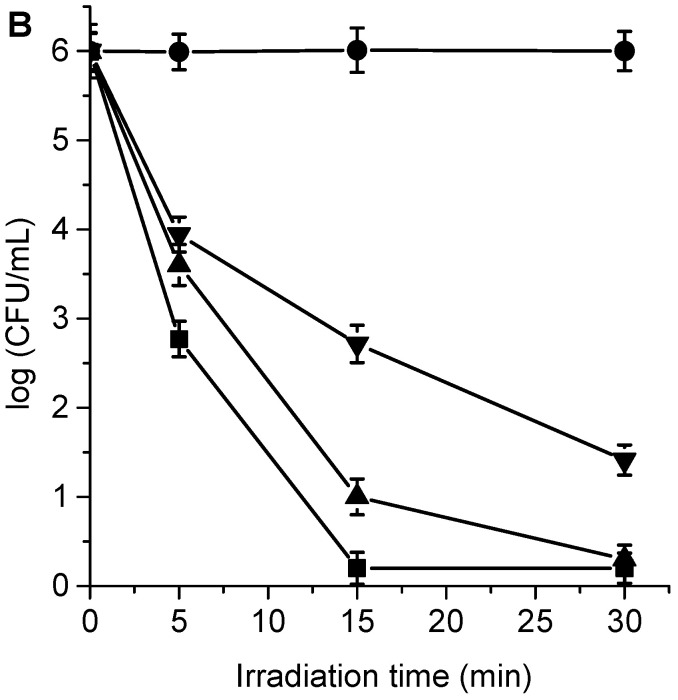
Survival of *C. albicans* (~10^6^ UFC/mL) treated with (**A**) 1 μM TPCF_16_ (△), 1 μM ZnTPCF_16_ (☐), 1 μM PdTPCF_16_ (▽), (**B**) 5 μM TPCF_16_ (▲), 5 μM ZnTPCF_16_ (■), and 5 μM PdTPCF_16_ (▼) for 30 min at 37 °C in the dark and irradiated with white light for different irradiation times. Control of *C. albicans* untreated with PS and irradiated (●).

The cytotoxic effect sensitized by TPCF_16_, ZnTPCF_16_, and PdTPCF_16_ was first evaluated in *C. albicans* cell suspensions in PBS. The cultures of yeast (~10^6^ UFC/mL) were incubated with 1 and 5 μM PS for 30 min in the dark at 37 °C and irradiated with white light for different periods of time (5, 15, and 30 min, which correspond to 27, 81, and 162 J/cm^2^, respectively). At these concentrations of chlorins, the viability of *C. albicans* was not affected by incubation in the dark (Figures S2 and S3). Furthermore, cell survival was not modified by irradiation of the culture without chlorins (Figure 5). Therefore, these control experiments confirm that the photoinactivation of *C. albicans* was caused by chlorin-induced photodynamic activity. As shown in Figure 5, photokilling of *C. albicans* was dependent on the chlorin derivative, PS concentration, and the irradiation times. Therefore, by modifying any of these variables, it is possible to change and control the degree of photoinactivation of *C. albicans*. When cultures were incubated with 1 μM PS and irradiated for 15 min, the photodynamic effect induced by PdTPCF_16_ and TPCF_16_ produced reductions of 2.8 and 3.0 log, respectively. In these conditions, ZnTPCF_16_ sensitized an inactivation of 3.7 log. Moreover, photokilling increased to 3.4 log for PdTPCF_16_ and 4.1 log for TPCF_16_, whereas viable *C. albicans* cells were not detected for ZnTPCF_16_ after 30 min of irradiation. For the three chlorins, the photoinactivation of fungal cells increased when cells were incubated with 5 μM PS, being more noticeable for TPCF_16_ and ZnTPCF_16_. Thus, a decrease of 5 log was found for cultures treated with TPCF_16_ upon an irradiation of 15 min, whereas viable cells were eliminated using ZnTPCF_16_. Furthermore, no cell survival was detected in presence of free-base chlorin and its Zn(II) complex after 30 min of irradiation, which represents a cell photoinactivation greater than 99.9996%. It was previously found that 5,10,15,20-tetrakis[4-(3-*N*,*N*-dimethylaminopropoxy)phenyl]chlorin (TAPC) was also an effective PS to inactivate *C. albicans* [36].

### 3.5. Photodynamic Mechanism in C. albicans Cells

**Figure 6:**
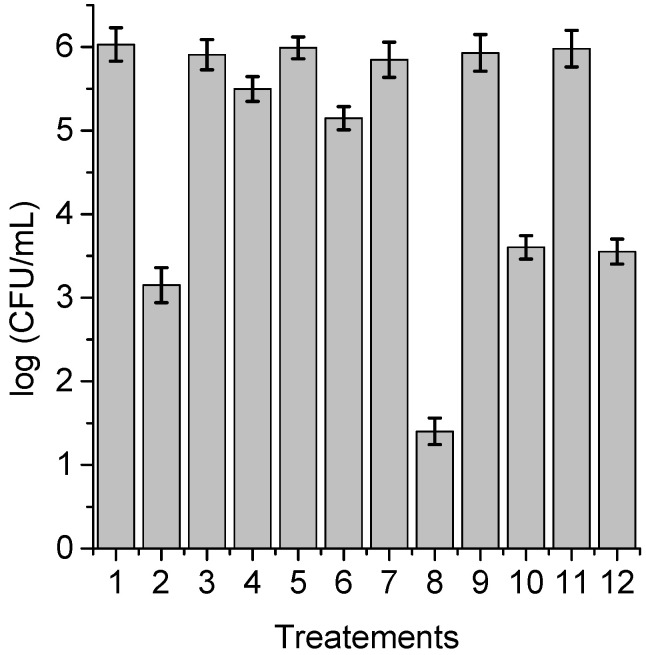
Survival of *C. albicans* (~10^6^ UFC/mL) treated with 1 μM TPCF_16_ for 30 min at 37 °C in the dark and irradiated with white light for 15 min; (1) cells; (2) cells treated with PS; (3) cells treated with 50 mM sodium azide; (4) cells treated with 50 mM sodium azide and PS; (5) cells treated with 50 mM DABCO; (6) cells treated with 50 mM DABCO and PS; (7) cells in D_2_O; (8) cells in D_2_O and treated with PS; (9) cells treated with 50 mM D-mannitol; (10) cells treated with 50 mM D-mannitol and PS; (11) cells treated with 50 mM cysteine and PS; (12) cells treated with 50 mM cysteine and PS.

**Figure 7:**
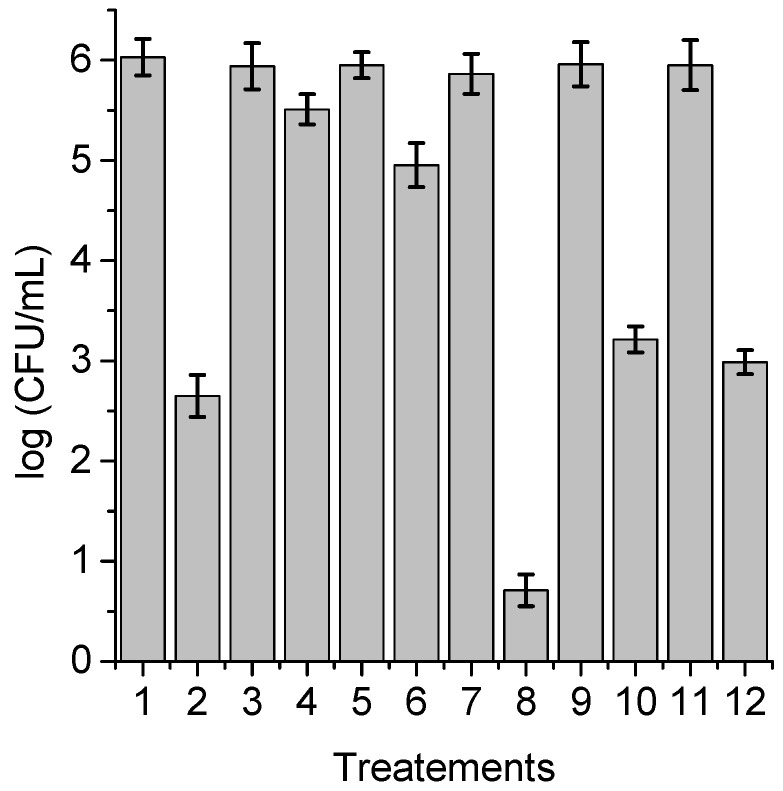
Survival of *C. albicans* (~10^6^ UFC/mL) treated with 1 μM ZnTPCF_16_ for 30 min at 37 °C in the dark and irradiated with white light for 15 min; (1) cells; (2) cells treated with PS; (3) cells treated with 50 mM sodium azide; (4) cells treated with 50 mM sodium azide and PS; (5) cells treated with 50 mM DABCO; (6) cells treated with 50 mM DABCO and PS; (7) cells in D_2_O; (8) cells in D_2_O and treated with PS; (9) cells treated with 50 mM D-mannitol; (10) cells treated with 50 mM D-mannitol and PS; (11) cells treated with 50 mM cysteine and PS; (12) cells treated with 50 mM cysteine and PS.

**Figure 8:**
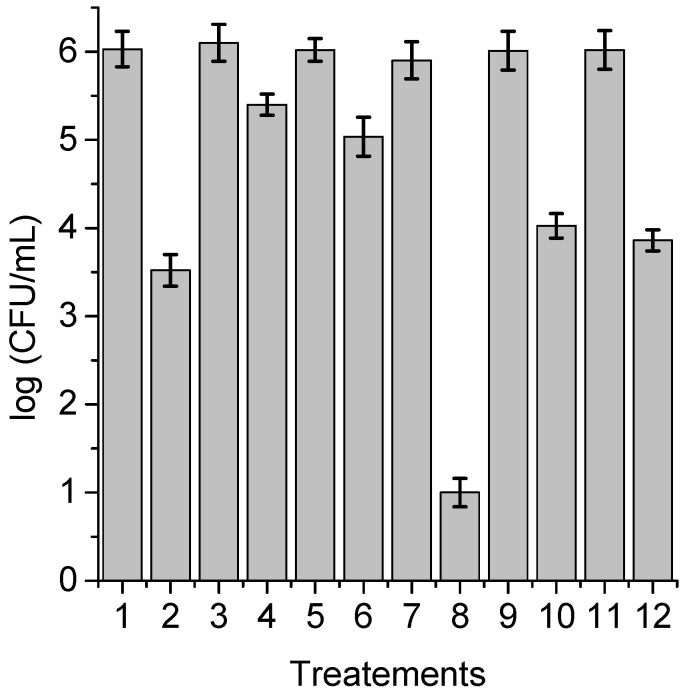
Survival of *C. albicans* (~10^6^ UFC/mL) treated with 1 μM PdTPCF_16_ for 30 min at 37 °C in the dark and irradiated with white light for 15 min; (1) cells; (2) cells treated with PS; (3) cells treated with 50 mM sodium azide; (4) cells treated with 50 mM sodium azide and PS; (5) cells treated with 50 mM DABCO; (6) cells treated with 50 mM DABCO and PS; (7) cells in D_2_O; (8) cells in D_2_O and treated with PS; (9) cells treated with 50 mM D-mannitol; (10) cells treated with 50 mM D-mannitol and PS; (11) cells treated with 50 mM cysteine and PS; (12) cells treated with 50 mM cysteine and PS.

With the purpose of obtaining information about the photodynamic mechanism of action sensitized by TPCF_16_, ZnTPCF_16_, and PdTPCF_16_ in *C. albicans* cell suspensions, PDI studies were carried out in the presence of ROS scavengers and D_2_O (Figure 6, Figure 7 and Figure 8). Cultures were first treated with the additives for 30 min at 37 °C in the dark and then with 1 μM chlorin. Cell viability was not affected in yeast cultures incubated with 50 mM of these compounds in the dark and exposed for 15 min to white light in absence of PSs (Figure 6, Figure 7 and Figure 8, lines 3, 5, 9, and 11). Furthermore, no toxicity was found for the irradiated cell suspensions in D_2_O (Figure 6, Figure 7 and Figure 8, line 7). In these experiments, a PS concentration of 1 μM and 15 min of irradiation were chosen to produce a photoinactivation level of *C. albicans* of approximately 3 log and, thus, to be able to visualize the effect produced by the additives or the D_2_O medium. The PDI results are shown in Figure 6, Figure 7 and Figure 8. Sodium azide and DABCO were used as quenchers of O_2_(^1^Δ_g_) [40,41]. With both additives, a reduction in photoinactivation was found in PDI treatments of *C. albicans*. The azide ions produced a reduction of about 2.5 log in the inactivation of yeast cells treated with TPCF_16_ and ZnTPCF_16_, (Figure 6 and Figure 7, line 4), whereas this effect was slightly lower with PdTPCF_16_, reaching 2 log of protection (Figure 8, line 4). Similar results were found for cultures incubated with DABCO and chlorins (Figure 6, Figure 7 and Figure 8, line 6), although with a decrease in inactivation somewhat less than that produced by sodium azide. Therefore, azide ions and DABCO produced a significant decrease in chlorin-sensitized photodynamic action by quenching O_2_(^1^Δ_g_). To confirm the involvement of a type II mechanism, D_2_O was used instead of water in order to increase the O_2_(^1^Δ_g_) lifetime [42]. PDI treatments of *C. albicans* cell suspensions in D_2_O with chlorin produced a significant increase in yeast photoinactivation relative to cells in PBS (Figure 6, Figure 7 and Figure 8, line 8). The greatest effect was observed for the metalated chlorins, producing an increase of about 2 log in cell inactivation. These results also suggest the participation of O_2_(^1^Δ_g_) in the photodynamic pathway that produces cell death. On the other hand, D-mannitol and L-cysteine can act as radical scavengers, and thus these compounds can be used as inhibitors of the type I photoprocess [43,44]. For all three chlorins, the addition of D-mannitol produced about 0.5 log in *C. albicans* cell protection (Figure 6, Figure 7 and Figure 8, line 10). Comparable behavior was found in yeast cultures when L-cysteine was used as a free radical scavenger (Figure 6, Figure 7 and Figure 8, line 12). Therefore, the presence of D-mannitol and L-cysteine in *C. albicans* cell suspensions produce virtually no significant changes in yeast photoinactivation, indicating a negligible contribution from a type I photoprocess.

In general, this is the behavior mainly found for PSs derived from tetrapyrrole macrocycles [45]. Analogous results were observed for the photokilling of *C. albicans* induced by porphyrins bearing cationic substituents or precursor groups of positive charges [24,41,46]. In addition, a type II photoreaction was found to be involved in the photodamage of yeast cells sensitized by TAPC free-base chlorin [28]. Therefore, the photodynamic action sensitized by chlorins in the present study involves the formation of O_2_(^1^Δ_g_) as the main ROS that leads to the death of *C. albicans* cells.

### 3.6. Photoinactivation of C. albicans Cells under Growth Conditions

**Figure 9:**
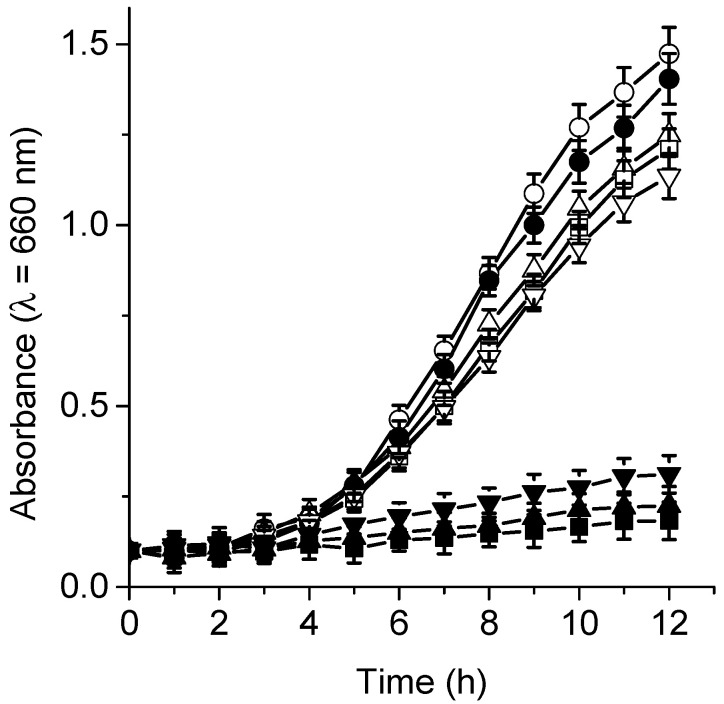
Growth curves of *C. albicans* treated with 5 μM TPCF_16_ (▲), ZnTPCF_16_ (■), and PdTPCF_16_ (▼) and irradiated with white light for different irradiation times at 37 °C. Controls: cells untreated with PS and irradiated (●), *C. albicans* untreated with PS in the dark (◯), cells treated with 5 μM TPCF_16_ (△), ZnTPCF_16_ (☐), and PdTPCF_16_ (▽) in the dark.

The photocytotoxic effect sensitized by TPCF_16_, ZnTPCF_16_, and PdTPCF_16_ was tested on the growth of *C. albicans* cultures in Sabouraud broth. These experiments were used to evaluate the ability of these compounds to photoinactivate *C. albicans* cells when the yeast cultures are in an appropriate medium for their growth [26]. Therefore, 5 μM chlorin was added to fresh cultures of *C. albicans* in the growth medium, and the cells were subsequently irradiated with white light at 37 °C. Figure 9 shows the photodynamic activity induced by the chlorin on yeast cell growth. Untreated *C. albicans* cells irradiated or incubated with PS in the dark behaved similarly to the control in the dark. However, cell growth did not occur when chlorin-containing *C. albicans* cultures were irradiated with white light. Although the photodynamic effect probably acts in the early part of the growth curve, it was previously demonstrated that these chlorin derivatives were highly photostable in cellular cultures [18].

In similar tests, a delay was previously found for the growth of *C. albicans* cells treated with 5 μM tetracationic porphyrin derivatives, although cell growth was not completely stopped [23,47]. Using the same concentration, 5,10,15,20-tetrakis[4-(3-*N*,*N*-dimethylaminopropoxy)phenyl]porphyrin (TAPP) was able to produce a reduction in the growth of *C. albicans* [26]. Furthermore, growth of 5 µM TAPC-treated cultures was retarded when the yeast cells were continuously irradiated with white light [28].

### 3.7. Photoinactivation of C. albicans Pseudohyphae

**Figure 10:**
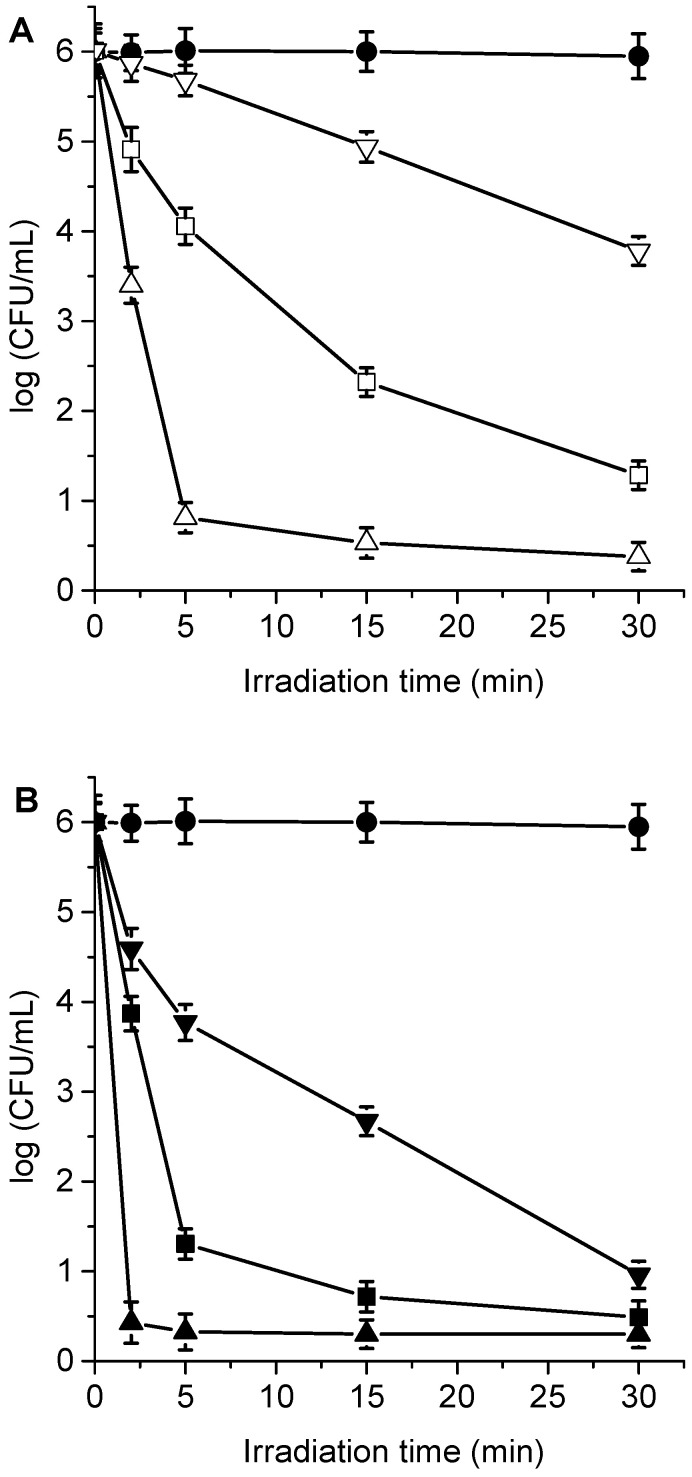
Survival of *C. albicans* pseudohyphae (~10^6^ UFC/mL) treated with (**A**) 1 μM TPCF_16_ (△), 1 μM ZnTPCF_16_ (☐), 1 μM PdTPCF_16_ (▽), (**B**) 5 μM TPCF_16_ (▲), 5 μM ZnTPCF_16_ (■), and 5 μM PdTPCF_16_ (▼) for 30 min at 37 °C in the dark and irradiated with white light for different irradiation times. Control of *C. albicans* pseudohyphae untreated with PS and irradiated (●).

*C. albicans* has the ability to change its morphological state from a yeast-like state, initially developing a germ tube and going through different elongated forms, called pseudohyphae, until finally forming hyphae [48,49]. This dimorphism allows certain fungi to switch between the unicellular yeast morphology to the multicellular filamentous form of hyphae in response to environmental changes. At the microbiological level, dimorphism is considered an important virulence factor in this yeast because it allows it to later colonize the mucosal tissues [50]. It is for this reason that the photodynamic activity induced by TPCF_16_, ZnTPCF_16_, and PdTPCF_16_ was evaluated in *C. albicans* pseudohyphae suspended in PBS. The formation of the dimorphic state of *C. albicans* was induced in yeast cultures suspended in HS for 4 h at 37 °C. The development of pseudohyphae was verified with optical microscopy [28]. *C. albicans* pseudohyphae cell suspensions (~10^6^ CFU/mL) in PBS were treated with 1 and 5 μM chlorin for 30 min in the dark at 37 °C. Figure 10 shows the survivals of pseudohyphae after different times of irradiations (2, 5, 15, and 30 min) with white light. The viability of the pseudohyphae was not modified by irradiation without incubation with chlorin. Furthermore, no toxicity was found for cells incubated with 5 μM chlorin in the dark for 30 min (Figures S4 and S5). Consequently, the photokilling of pseudohyphae upon irradiation with white light was caused by the photosensitizing action of the chlorins. As can be observed in Figure 10, 1 μM TPCF_16_ was effective to photoinactivate *C. albicans* pseudohyphae suspended in PBS, producing a decrease in cell viability of over 5 log after 5 min of irradiation. In addition, 5 μM free-base chlorin was able to eliminate yeast upon an irradiation of 2 min. At the highest concentration used, complexes with Zn(II) and Pd(II) were also active PSs to inactivate pseudohyphae, achieving a decrease in cell survival greater than 5 log after 30 min exposure to white light.

In previous investigations, light-activated antimicrobial agent toluidine blue O (TBO) was able to kill the hyphal form of *C. albicans*, resulting in a 5.2 log reduction in its viability [51]. In addition, the irradiation of germ tubes of *C. albicans* incubated with Photofrin produced significant cell damage [52]. In the present work, photokilling of pseudohyphae sensitized by TPCF_16_ and ZnTPCF_16_ was more effective than previously observed results for TAPC-treated cultures, whereas the photoinactivating capacity of PdTPCF_16_ was similar to the latter free-base chlorin [28].

### 3.8. Photokilling of C. albicans Biofilms

**Figure 11:**
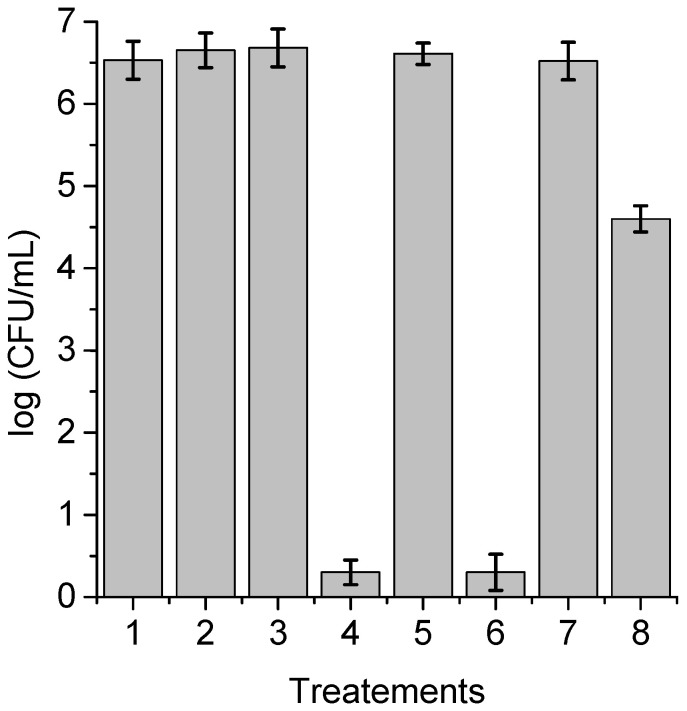
Survival of *C. albicans* incubated with 5 μM PS for 18 h at 37 °C in the dark and irradiated with white light for 60 min; (1) cells in the dark; (2) irradiated cells; (3) cells treated with TPCF_16_ in the dark; (4) irradiated cells treated with TPCF_16_; (5) cells treated with ZnTPCF_16_ in the dark; (6) irradiated cells treated with ZnTPCF_16_; (7) cells treated with PdTPCF_16_ in the dark; (8) irradiated cells treated with PdTPCF_16_.

In immunosuppressed or medically compromised individuals, *C. albicans* infection can lead to the establishment of candidiasis, which can manifest as a superficial or invasive disease [53]. In this sense, most of the nosocomial septicemias found derive from intravascular and urinary catheters, heart valves, and silicone prostheses, among others. Some of the main reasons that make this type of infection possible are the capacity for filamentous growth, the formation of biofilms, and the production of extracellular material together with the modification of the structure of their cell walls [54]. Taking this into account, the photoinactivation of *C. albicans* sensitized by TPCF_16_, ZnTPCF_16_, and PdTPCF_16_ was evaluated in biofilms grown on PVC discs, which is one of the materials commonly used in the manufacture of catheters [55]. After the adhesion step, the cultures were treated with 5 μM chlorin in Sabouraud broth supplemented with 7% FBS for 18 h at 37 °C in the dark during biofilm proliferation. The survival rates of *C. albicans* after PDI treatments are indicated in Figure 11. No toxicity was observed for biofilms irradiated for 60 min without PS, nor for those treated with chlorin and kept in the dark. The photodynamic effect mediated by TPCF_16_ and ZnTPCF_16_ produced the eradication of *C. albicans* cells in the biofilms upon 60 min of irradiation. This denotes a greater than 6 log decrease in yeast survival, which represents 99.9997% photoinactivation. In contrast, a lower photokilling capacity was found with PdTPCF_16_, which induced a 2 log reduction in the viability of *C. albicans*. Despite the fact that PdTPCF_16_ presented the highest value of Φ_Δ_, the photoinactivation induced by the Pd(II) complex was lower than that produced by both the Zn(II) complex and even the free-base chlorin. It was previously found that Pd(II) tetrapyrrole macrocycle complexes can form aggregates in aqueous media, thus decreasing photodynamic activity and the ability to interact with biological media [30].

To verify the efficacy of the photodynamic action sensitized by TPCF_16_ and ZnTPCF_16_, after the PDI treatments, the PVC discs were deposited on a Sabouraud agar plate and incubated for 48 h at 37 °C in the dark (Figure S6). After that, significant proliferation of viable *C. albicans* cells was observed in controls of the biofilms kept in the dark, the biofilms irradiated for 60 min, and the biofilms treated with 5 μM chlorin in the dark. In contrast, no cell growth was found on the PVS discs treated with 5 μM TPCF_16_ or ZnTPCF_16_ after an irradiation time of 60 min. Furthermore, similar results were obtained by placing the PVC discs in Sabouraud broth after 48 h of incubation at 37 °C in the dark (Figure S7). The characteristic turbidity of yeast cell growth was observed in the test tubes containing the PVC discs with the different biofilm controls (Figure S7A–C and E). However, for the PVC discs subjected to the PDI treatments induced by 5 μM TPCF_16_ or ZnTPCF_16_, the tubes did not show growth or development of *C. albicans* in the liquid medium (Figure S7D,F). These results confirm the efficiency of free-base chlorin and its complex with Zn(II) to eradicate *C. albicans* biofilms.

The effects of PDI were previously investigated using TBO on the viability of biofilms produced by *C. albicans* at different stages of development [56]. The photodynamic activity inhibited biofilm formation, and the PDI treatment was able to decrease the survival of yeast cells and filamentous form in the biofilm in both early and mature biofilms. Furthermore, the photoinactivation produced by 5-aminolevulinic acid (ALA) was investigated on *Candida albicans* biofilms [57,58]. The presence of ALA showed a significant increase of protoporphyrin IX (PpIX) in the biofilms. The metabolic activity of *C. albicans* biofilms treated with ALA confirmed the inhibition efficacy. Cells in *C. albicans* biofilms were 74.45% inhibited upon radiation at 300 J/cm^2^ [57]. Degraded cytoplasmic content, nuclear condensation, and mitochondrial swelling were observed after PDI treatments [58]. Moreover, a panel of porphyrins was evaluated for PDI applications to control the growth of *C. albicans* [59]. In particular, monocationic diaryl-porphyrins were effective PSs to kill the *C. albicans* cells and inhibit biofilm formation. The tetracationic metalloporphyrin Zn(II) *meso*-tetrakis(*N*-*n*-hexylpyridinium-2-yl)porphyrin (ZnTnHex-2-PyP^4+^) was also investigated as a PS to inactivate yeasts and biofilms of *C. albicans* using a blue light-emitting diode [60]. At 0.8 μM and 4.3 J/cm^2^ light doses, PDI-treated biofilms showed decreases in cell viability and structural alterations with reduced hyphae. The photodynamic efficiency of a formulation composed of five cationic porphyrins and its combined effect with potassium iodide was tested on a large spectrum of microorganisms [61]. This combination was efficient in the destruction of *C. albicans* biofilms. In the present study, TPCF_16_ and ZnTPCF_16_ showed interesting properties as potential phototherapeutic agents for the efficient elimination of *C. albicans* yeast, pseudohyphae, and biofilms.

## 4. Conclusions

The PDI of yeasts is an interesting new therapy to treat localized fungal infections. In the search for new PSs, in this work we evaluated the photodynamic activity of a free-base chlorin and its complexes with Zn(II) and Pd(II) in *C. albicans* under different forms of cell cultures. These compounds absorbed intensely in the visible region and produced O_2_(^1^Δ_g_) efficiently, mainly in the complexes of free-base chlorin with Zn(II) and Pd(II) due to the heavy-atom effect. Mainly, TPCF_16_ and ZnTPCF_16_ were able to kill *C. albicans* planktonic cells at low PS concentrations and short irradiation periods. The photodynamic mechanism of action involved the formation of O_2_(^1^Δ_g_) as the main ROS that causes the inactivation of *C. albicans* cells. Moreover, the growth of *C. albicans* was suppressed by the photodynamic effect produced by these chlorins. The morphological plasticity of *C. albicans* was a determining factor of virulence because the pseudohyphal form has an important role in the infection process. The free-base chlorin and its Zn(II) complex were also effective in eliminating *C. albicans* pseudohyphae suspended in PBS. On the other hand, *C. albicans* can form biofilms on solid surfaces in the environment and within mammalian hosts in infections. Biofilms related to medical devices are of great clinical importance due to their high resistance to conventional antifungal agents for hospital use. When chlorins were incorporated during the proliferation stage, the photodynamic effect induced by TPCF_16_ and ZnTPCF_16_ was able to eradicate the *C. albicans* biofilm. Therefore, these results indicated that TPCF_16_ and ZnTPCF_16_ are interesting potential phototherapeutic agents to eliminate *C. albicans* with different morphologies and growth forms.

## Figures and Tables

**Table 1:** Spectroscopic characteristics of TPCF_16_, ZnTPCF_16_, and PdTPCF_16_ in DMF.

PS	λ^Soret^ (nm)	ε^Soret a^	λ^QI^ (nm)	ε^QI a^	λ_em_ (nm)	Φ_F_ ^b^
TPCF_16_	408	1.5 × 10^5^	651	3.5 × 10^4^	653	0.11 ± 0.01 ^c^
ZnTPCF_16_	418	1.7 × 10^5^	621	2.9 × 10^4^	626	0.040 ± 0.002
PdTPCF_16_	405	1.6 × 10^5^	602	5.7 × 10^4^	-	-

^a^ Molar absorption coefficient (Lmol^−1^cm^−1^), ^b^ fluorescence quantum yield, ^c^ from ref. [18].

**Table 2:** Photodynamic properties of TPCF_16_, ZnTPCF_16_, and PdTPCF_16_ in DMF.

PS	*k*_obs_^DMA^ (s^−1^) ^a^	Φ_Δ_ ^b^
TPCF_16_	-	0.34 ± 0.02 ^c^
ZnTPCF_16_	(5.81 ± 0.05) × 10^−4^	0.71 ± 0.03 ^d^
PdTPCF_16_	(6.15 ± 0.07) × 10^−4^	0.75 ± 0.04 ^d^

^a^ Observed rate constants for the photo-oxidation reaction of DMA, ^b^ quantum yield of O_2_(^1^Δ_g_) production, ^c^ from ref. [18], ^d^ using MB as a reference: *k*_obs_ = (4.26 ± 0.04) × 10^−4^ s^−1^, Φ_Δ_ = 0.52 [22].

## Data Availability

Not applicable.

## Supplementary Materials

The following are available online at https://www.mdpi.com/article/10.3390/antibiotics12010105/s1. Materials; Instrumentation; Figure S1: Absorption spectral changes during the photo-oxidation of DMA sensitized by ZnTPCF_16_ and PdTPCF_16_ in DMF at different irradiation times; Figure S2: Survival of *C. albicans* treated with 1 μM TPCF_16_, ZnTPCF_16_, and PdTPCF_16_ for 30 min at 37 °C in the dark and kept in the dark for different amounts of time; Figure S3: Survival of *C. albicans* treated with 5 μM TPCF_16_, ZnTPCF_16_, and PdTPCF_16_ for 30 min at 37 °C in the dark and kept in the dark for different amounts of time; Figure S4: Survival curves of *C. albicans* pseudohyphae incubated with 1 μM TPCF_16_, ZnTPCF_16_, and PdTPCF_16_ for in PBS for 30 min at 37 °C in dark and kept in the dark for different amounts of time; Figure S5: Survival curves of *C. albicans* pseudohyphae incubated with 5 μM TPCF_16_, ZnTPCF_16_, and PdTPCF_16_ for in PBS for 30 min at 37 °C in dark and kept in the dark for different amounts of time; Figure S6: Photographs of PVC discs deposited on a Sabouraud agar plate and incubated for 48 h at 37 °C in the dark, control of *C. albicans* biofilm in the dark, control of *C. albicans* biofilm irradiated for 60 min with white light, *C. albicans* biofilm treated with 5 μM TPCF_16_ in the dark, *C. albicans* biofilm treated with 5 μM TPCF_16_ irradiated for 60 min with white light, (E) *C. albicans* biofilm treated with 5 μM ZnTPCF_16_ in the dark, *C. albicans* biofilm treated with 5 μM ZnTPCF_16_ irradiated for 60 min with white light; Figure S7: Photographs of PVC discs in Sabouraud broth and incubated for 48 h at 37 °C in the dark, control of *C. albicans* biofilm in the dark, control of *C. albicans* biofilm irradiated for 60 min with white light, *C. albicans* biofilm treated with 5 μM TPCF_16_ in the dark, *C. albicans* biofilm treated with 5 μM TPCF_16_ irradiated for 60 min with white light, (E) *C. albicans* biofilm treated with 5 μM ZnTPCF_16_ in the dark, *C. albicans* biofilm treated with 5 μM ZnTPCF_16_ irradiated for 60 min with white light. References [18,28,30,34,62,63,64] are cited in the supplementary materials.
