# Supplementary material for: Photoinactivation of Planktonic Cells, Pseudohyphae, and Biofilms of Candida albicans Sensitized by a Free-Base Chlorin and Its Metal Complexes with Zn(II) and Pd(II)
Source: Antibiotics (Basel). 2023 Jan 6;12(1):105. doi: 10.3390/antibiotics12010105 (PMC9854949; doi:10.3390/antibiotics12010105)
Supplement: Supplementary file 1 [file antibiotics-12-00105-s001.zip › antibiotics-2136641-supplementary.pdf]

Supplementary Materials

Photoinactivation of Planktonic Cells, Pseudohyphae, and Biofilms of *Candida albicans* Sensitized by a Free-Base Chlorin and Its Metal Complexes with Zn(II) and Pd(II)

Paula V. Cordero[1], María G. Alvarez[1], Edwin J. Gonzalez Lopez[1], Daniel A. Heredia[1] and Edgardo N. Durantini[1]*

[1] IDAS-CONICET, Departamento de Química, Facultad de Ciencias Exactas, Físico-Químicas y Naturales, Universidad Nacional de Río Cuarto, Ruta Nacional 36 Km 601, X5804BYA Río Cuarto, Córdoba, Argentina.

Table of Contents

1. Materials Page S2

2. Instrumentation Page S3

3. Scheme and figures Page S4

4. References Page S7

* Correspondence: edurantini@exa.unrc.edu.ar

1. Materials

Compounds were obtained from Sigma-Aldrich (Milwaukee, WI, USA), which were used without further purification. Organic solvents (GR grade) from Merck (Darmstadt, Germany) were distilled and maintained on molecular sieves. Ultrapure water was obtained from a Labconco (Kansas City, MO, USA) equipment model 90901-01. Silica gel thin-layer chromatography (TLC) plates (250 microns) were acquired from Analtech (Newark, DE, USA) and silica gel 60 (0.040-0.063 mm, 230-400 mesh) from Merck (Darmstadt, Germany). Sabouraud glucose broth and agar from Britania (Buenos Aires, Argentina) were used in yeast cultures. Microtiter plates (96-well and 24-wells) were acquired to Deltalab (Barcelona, Spain). 5,10,15,20-Tetrakis(4-methoxyphenyl)porphyrin (TMP) and 5,10,15,20-tetrakis[4-(2-(dimethylamino)ethoxy)-2,3,5,6-tetrafluorophenyl]-2,3-[methano(*N*-methyl)iminomethano]chlorin ($TPCF_{16}$) were synthesized as reported [1,2]. Zn(II) 5,10,15,20-Tetrakis[4-(2-(dimethylamino)ethoxy)-2,3,5,6-tetrafluorophenyl]-2,3-[methano(*N*-methyl)iminomethano]chlorin ($ZnTPCF_{16}$) and Pd(II) 5,10,15,20-tetrakis[4-(2-(dimethylamino)ethoxy)-2,3,5,6-tetrafluorophenyl]-2,3-[methano(*N*-methyl)iminomethano]chlorin ($PdTPCF_{16}$) were prepared according to previously described methods [3,4]. $ZnTPCF_{16}$ was prepared dissolving 20 mg (0.015 mmol) of $TPCF_{16}$ in 7 mL of dichloromethane. Then, 3 mL of a saturated solution of Zn(II) acetate in methanol was added to the solution. The mixture was stirred for 12 h under argon atmosphere at room temperature. After that, the solution was treated with water (30 mL) and the organic phase was extracted with three portions of dichloromethane (20 mL each). The organic solvents were evaporated under reduced pressure and the crude product was purified by column chromatography (silica gel, dichloromethane/methanol, 9:1) to afford 18 mg (88%) of pure $ZnTPPF_{16}$; MS-ESI [m/z]: 1370.3487 (1370.3491 calculated for $[M+H]^+$, M = $C_{63}H_{55}F_{16}N_9O_4Zn$). $PdTPCF_{16}$ was obtained dissolving 20 mg (0.015 mmol) of $TPCF_{16}$ in 10 mL of *N*,*N*-dimethylformamide (DMF). After that, Pd(II) chloride (50 mg,0.28 mmol) was added to the solution and the mixture was stirred for 6 h under argon atmosphere at reflux. The solvent was evaporated under reduced pressure and the crude product was purified by column chromatography (silica gel,

dichloromethane/methanol, 9:1) to afford 16 mg (75%) of pure $PdTPPF_{16}$; MS-ESI [m/z]: 1412.3229 (1412.3235 calculated for $[M+H]^+$, M = $C_{63}H_{55}F_{16}N_9O_4Pd$).

2. Instrumentation

Mass spectra were recorded on a Bruker micrOTOF-QII (Bruker Daltonics, Billerica, MA, USA) equipped with an ESI source (ESI-MS). UV-visible absorption spectra were carried out on a Shimadzu UV-2401PC spectrometer (Shimadzu Corporation, Tokyo, Japan). Fluorescence emission spectra were performed on a Spex FluoroMax spectrofluorometer (Horiba Jobin Yvon Inc, Edison, NJ, USA). A Radiometer Laser Mate-Q (Coherent, Santa Clara, CA, USA) was used to determine the light fluence rates. Steady-state photolysis of 9,10-dimethylanthracene (DMA) were performed with a Cole-Parmer illuminator 41720-series (150 W halogen lamp, Cole-Parmer, Vernon Hills, IL, USA) in combination with a high intensity grating monochromator (Photon Technology Instrument, Birmingham, NJ, USA) [5]. This arrangement produces a light fluence rate of 1.1 mW/cm^2 at 610 ± 6 nm. Cell suspensions were irradiated with a Novamat 130 AF (Braun Photo Technik, Nürnberg, Germany) projector containing a 150 W lamp. A 2.5 cm glass cuvette filled with water without circulation was used to remove the heat from the lamp. A wavelength range between 350 and 800 nm was selected by optical filters. The projector was placed vertically with the light beam focused on the 96-well microtiter plate lid, producing a fluence rate of 90 mW/cm^2 [6,7].

3. Scheme and figures

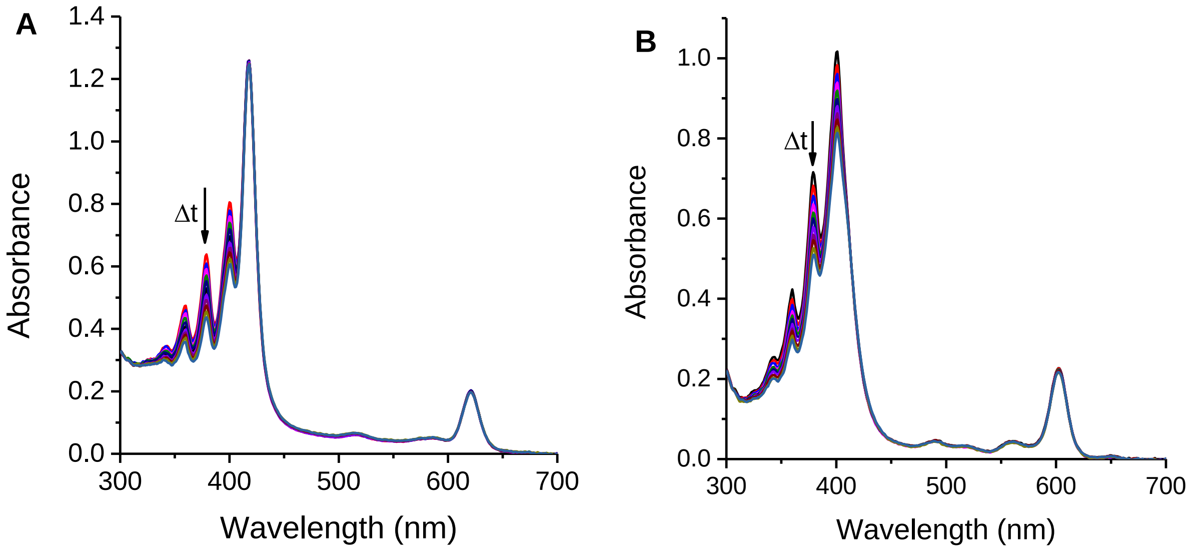

Figure S1. Absorption spectral changes during the photooxidation of DMA sensitized by (A) $ZnTPCF_{16}$ and (B) $PdTPCF_{16}$ in DMF at different irradiation times (Δt = 60 s), λ_{irr} = 610 nm.

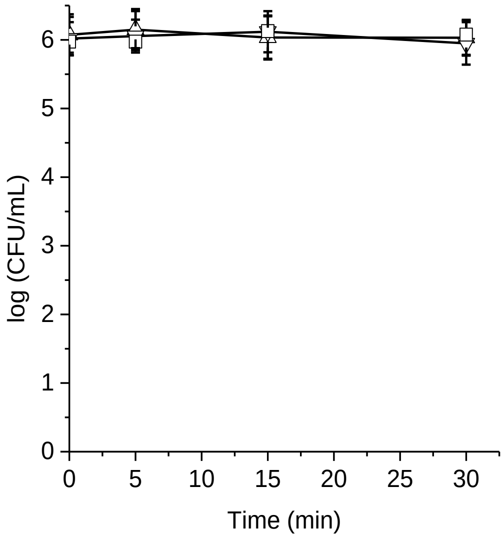

Figure S2. Survival of *C. albicans* (~10^6 UFC/mL) treated with 1 μM $TPCF_{16}$ (△), $ZnTPCF_{16}$ (□) and $PdTPCF_{16}$ (▽) for 30 min at 37 ºC in the dark and kept in the dark for different times.

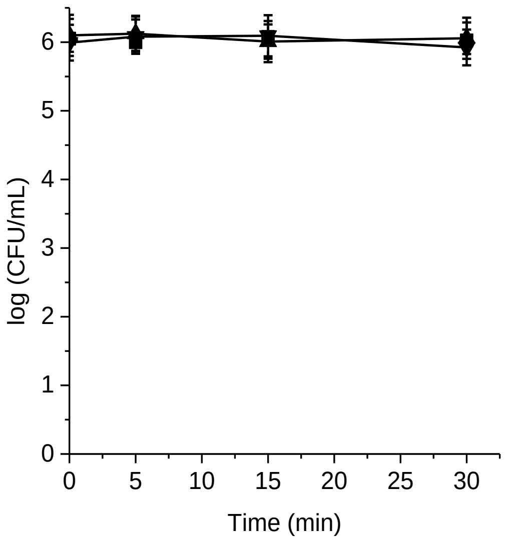

Figure S3. Survival of *C. albicans* (~10^6 UFC/mL) treated with 5 μM $TPCF_{16}$ (▲), $ZnTPCF_{16}$ (■) and $PdTPCF_{16}$ (▼) for 30 min at 37 ºC in the dark and kept in the dark for different times.

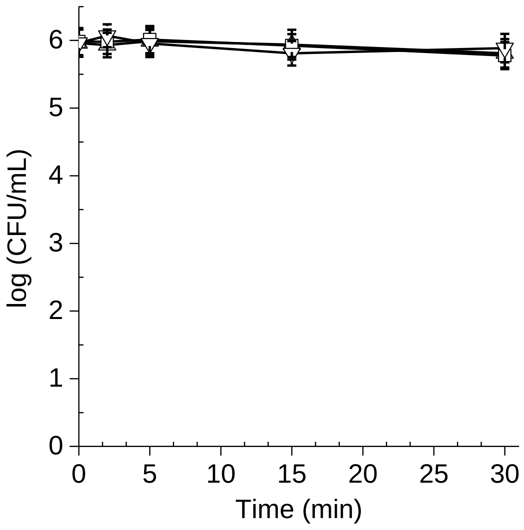

Figure S4. Survival curves of *C. albicans* pseudohyphae (~10^6 CFU/mL) incubated with 1 μM $TPCF_{16}$ (△), $ZnTPCF_{16}$ (□) and $PdTPCF_{16}$ (▽) for in PBS for 30 min at 37 ºC in dark and kept in the dark for different times.

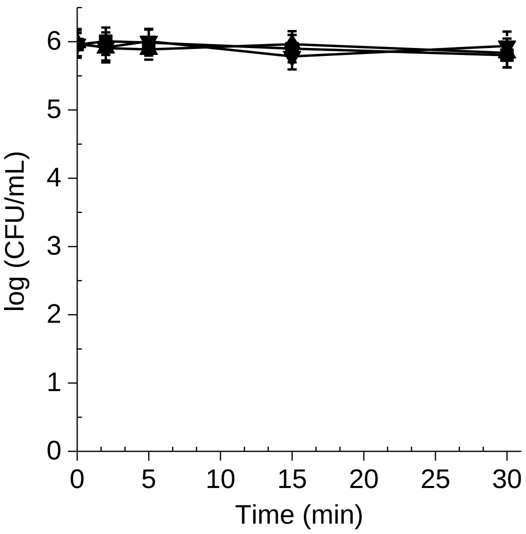

Figure S5. Survival curves of *C. albicans* pseudohyphae (~10^6 CFU/mL) incubated with 5 μM $TPCF_{16}$ (▲), 5 μM $ZnTPCF_{16}$ (■) and 5 μM $PdTPCF_{16}$ (▼) for in PBS for 30 min at 37 ºC in dark and kept in the dark for different times.

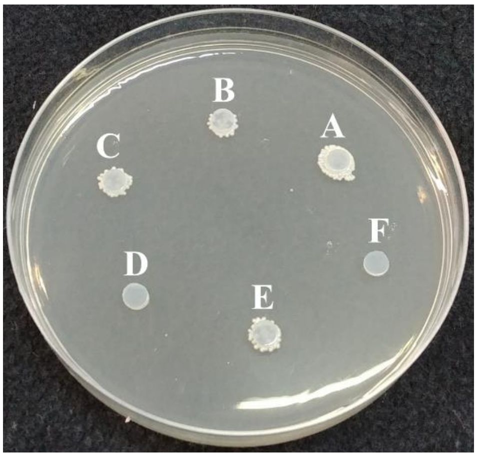

Figure S6. Photographs of PVC discs deposited on a Sabouraud agar plate and incubation for 48 h at 37 ºC in the dark; (A) control of *C. albicans* biofilm in the dark, (B) control of *C. albicans* biofilm irradiated for 60 min with white light (90 mW/cm^2), (C) *C. albicans* biofilm treated with 5 μM $TPCF_{16}$ in the dark, (D) *C. albicans* biofilm treated with 5 μM $TPCF_{16}$ irradiated for 60 min with white light (90 mW/cm^2), (E) *C. albicans* biofilm treated with 5 μM $ZnTPCF_{16}$ in the dark, (F) *C. albicans* biofilm treated with 5 μM $ZnTPCF_{16}$ irradiated for 60 min with white light (90 mW/cm^2).

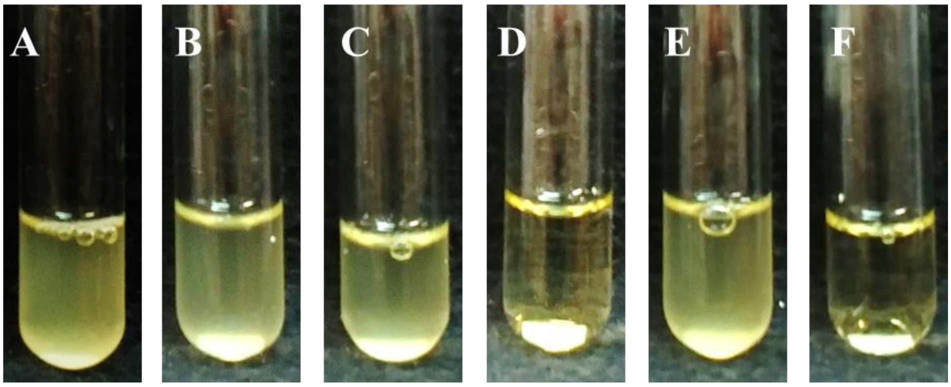

Figure S7. Photographs of PVC discs in Sabouraud broth and incubation for 48 h at 37 ºC in the dark, (A) control of *C. albicans* biofilm in the dark, (B) control of *C. albicans* biofilm irradiated for 60 min with white light (90 mW/cm^2), (C) *C. albicans* biofilm treated with 5 μM $TPCF_{16}$ in the dark, (D) *C. albicans* biofilm treated with 5 μM $TPCF_{16}$ irradiated for 60 min with white light (90 mW/cm^2), (E) *C. albicans* biofilm treated with 5 μM $ZnTPCF_{16}$ in the dark, (F) *C. albicans* biofilm treated with 5 μM $ZnTPCF_{16}$ irradiated for 60 min with white light (90 mW/cm^2).

4. References

1. Milanesio, M. E.; Alvarez, M. G.; Yslas, E. I.; Borsarelli, C. D.; Silber, J. J.; Rivarola, V.; Durantini, E. N. Photodynamic studies of metallo 5,10,15,20-tetrakis(4-methoxyphenyl) porphyrin: photochemical characterization and biological consequences in a human carcinoma cell line. *Photochem. Photobiol.* **2001**, *74*, 14-21.

2. Heredia, D. A.; Durantini, J. E.; Ferreyra, D. D.; Reynoso, E.; Gonzalez Lopez, E. J.; Durantini, A. M.; Milanesio, M. E.; Durantini, E. N. Charge density distribution effect in pyrrolidine-fused chlorins on microbial uptake and antimicrobial photoinactivation of microbial pathogens. *J. Photochem. Photobiol. B: Biol.* **2021**, *225*, 112321.

3. Milanesio, M. E.; Alvarez, M. G.; Bertolotti, S. G.; Durantini; E. N. Photophysical characterization and photodynamic activity of metallo 5-(4-(trimethylammonium)phenyl)-10,15,20-tris(2,4,6-

trimethoxyphenyl)porphyrin in homogeneous and biomimetic media. *Photochem. Photobiol. Sci.*, **2008**, *7*, 963-972.

4. Obata, M.; Hirohara, S.; Tanaka, R.; Kinoshita, I.; Ohkubo, K.; Fukuzumi, S.; Tanihara, M.; Yano, S. *In vitro* heavy-atom effect of palladium(II) and platinum(II) complexes of pyrrolidine-fused chlorin in photodynamic therapy. *J. Med. Chem.* **2009**, *52*, 2747-2753.

5. Pérez, M. E.; Durantini, J. E.; Reynoso, E.; Alvarez, M. G.; Milanesio, M. E.; Durantini, E. N. Porphyrin-schiff base conjugates bearing basic amino groups as antimicrobial phototherapeutic agents. *Molecules* **2021**, *26*, 5877.

6. Cordero, P. V.; Ferreyra, D. D.; Pérez, M. E.; Alvarez, M. G.; Durantini, E. N. Photodynamic effect of 5,10,15,20-tetrakis[4-(3-*N*,*N*-dimethylaminopropoxy)phenyl]chlorin towards the human pathogen *Candida albicans* under different culture conditions. *Photochem* **2021**, *1*, 505-522.

7. Santamarina, S. C.; Heredia, D. A.; Durantini, A. M.; Durantini, E. N. Antimicrobial photosensitizing material based on conjugated Zn(II) porphyrins. *Antibiotics* **2022**, *11*, 91.
